# Who Uses Memory Strategies? Exploring the Influence of Cognitive Reserve on Use of Memory Strategies by Older Adults Referred for Neuropsychological Assessment

**DOI:** 10.1002/alz70857_105397

**Published:** 2025-12-24

**Authors:** Emily Q Wang, Jarod A Joshi, Catherine Bosyj, Ana Badal, Kristoffer Romero, Renée K Biss

**Affiliations:** ^1^ University of Windsor, Windsor, ON, Canada; ^2^ York University, North York, ON, Canada

## Abstract

**Background:**

In older age, the ability to learn and remember new information is influenced by a myriad of factors, including the use of internal memory strategies. It has been proposed that individual differences in cognitive reserve (CR) may be related to older adults’ spontaneous use of these strategies. The current study examined the relative importance and moderating effects of two CR proxies—educational attainment and crystallized intelligence—on older adults’ use of a highly effective memory strategy, semantic clustering—grouping words together based on semantic relationships—during a verbal list‐learning task.

**Method:**

This study analyzed data from an archival sample of 185 older adults (*n* = 83 with normal cognition, n = 102 with mild cognitive impairment [MCI]) referred for neuropsychological assessment at a geriatric hospital in Ontario, Canada. A series of hierarchical regression models and relative weight analyses were conducted to examine the effects of education and crystallized intelligence (WASI Vocabulary scores) on semantic clustering, immediate recall, and delayed recall performance on the Kaplan‐Baycrest Neurocognitive Assessment word lists subtest. A moderation analysis examined whether the relationship between semantic clustering and delayed recall was moderated by CR proxies.

**Result:**

Gender and English language background predicted semantic clustering. Semantic clustering strategy‐use accounted for additional variance in memory performance beyond the effects of demographic or reserve variables. While CR proxies did not significantly enhance the predictive value of any model, a moderation analysis found that crystallized intelligence was negatively associated with delayed recall performance in patients with MCI. Semantic clustering predicted delayed recall performance, and the effect was not moderated by education or crystallized intelligence.

**Conclusion:**

In a clinical sample of older adults presenting for neuropsychological assessment, women and native English speakers were more likely to use semantic clustering, independent of CR. The relationship between CR and memory performance is complex in a clinical setting, where patients may be assessed at different stages of disease progression. Semantic clustering appears to bolster memory performance regardless of an individual's existing level of CR, suggesting that teaching older adults with both low and high CR internal memory strategies may help to reduce everyday memory problems.

